# A Circadian Rhythm in both Complement Cascade (ComC) Activation and Sphingosine-1-Phosphate (S1P) Levels in Human Peripheral Blood Supports a Role for the ComC–S1P Axis in Circadian Changes in the Number of Stem Cells Circulating in Peripheral Blood

**DOI:** 10.1007/s12015-018-9836-7

**Published:** 2018-06-17

**Authors:** Marta Budkowska, Ewa Ostrycharz, Adrianna Wojtowicz, Zuzanna Marcinowska, Jarosław Woźniak, Mariusz Z. Ratajczak, Barbara Dołęgowska

**Affiliations:** 1 Department of Medical Analytics, Pomeranian Medical University, Al. Powstańców Wielkopolskich 72, 70-111 Szczecin, Poland; 2 Centre for Human Structural and Functional Research, Faculty of Physical Education and Health Promotion, University of Szczecin, ul. Narutowicza 17C, 70-240 Szczecin, Poland; 3 Institute of Mathematics, Department of Mathematics and Physics, University of Szczecin, Ul. Wielkopolska 15, 70-451 Szczecin, Poland; 4 Stem Cell Biology Program at the James Graham Brown Cancer Center, University of Louisville, Louisville, KY 40202 USA; 5 Department of Regenerative Medicine, Center for Preclinical Research and Technology, Warsaw Medical University, ul. Banacha 1B, 02-097 Warsaw, Poland; 6 Department of Microbiology, Immunology and Laboratory Medicine, Pomeranian Medical University, Al. Powstańców Wielkopolskich 72, 70-111 Szczecin, Poland

**Keywords:** Circadian rhythm, Complement cascade (ComC), Anaphylatoxins, Membrane attack complex (MAC), Sphingosine-1-phosphate (S1P)

## Abstract

The number of hematopoietic stem/progenitor cells (HSPCs) circulating in peripheral blood (PB) is regulated by a circadian rhythm, and more HSPCs circulate in PB in the morning hours than at night. Different mechanisms have been proposed that might regulate this process, including changes in tonus of β-adrenergic innervation of bone marrow (BM) tissue. Our group reported that in mice circadian changes in the number of HSPCs circulating in PB correlates with diurnal activation of the complement cascade (ComC) and that the mice deficient in C5 component of ComC (C5-KO mice) do not show circadian changes in the number of circulating HSPCs in PB. We also reported the existence of a gradient between PB and BM of a bioactive phosphosphingolipid, sphingosine-1-phosphate (S1P), which is a major PB chemottractant for BM-residing HSPCs. Based on these observations, we investigated activation of the ComC and the level of S1P in the PB of 66 healthy volunteers. We found that both ComC activation and the S1P level undergo changes in a circadian cycle. While the ComC becomes highly activated during deep sleep at 2 am, S1P becomes activated later, and its highest level is observed at 8 am, which precedes circadian egress of HSPCs from BM into PB. In sum, circadian activation of the ComC–S1P axis releases HSPCs from BM into PB.

## Introduction

A day–night (or light–dark) circadian rhythm (biological clock) is synchronized by a process that is coordinated by the pineal gland, which produces and releases melatonin into peripheral blood (PB) [1]. Thus, melatonin is a well-defined biochemical coordinator of circadian rhythms [2–5]. It is synthesized only in the dark (primarily during sleep), and therefore is at a much lower level in PB during the day, increasing at night between 2 am and 4 am [1].

Several biological processes in the body undergo circadian changes caused by i) the release of relevant hormones (e.g., the abovementioned melatonin and adrenal gland steroids), ii) the tonus in innervation of tissues by the sympathetic and parasympathetic autonomic nerve fibers, iii) the sleeping–waking rhythm, and iv) the effect of light on the organism [1, 6]. Perturbation of the normal circadian rhythm may lead to organ dysfunction, accelerated atherosclerosis, and even neoplasm [4].

Our group became interested in circadian changes in the levels of HSPCs circulating in PB as part of a process of circadian rhythm-regulated mobilization of these cells from BM into PB [7]. The number of HSPCs circulating in PB is two-fold higher in the morning hours than at night [8–13]. Based on our observation that innate immunity and, in particular, the complement cascade (ComC) are major triggers for the egress of HSPCs from BM [7, 14–16], we performed mobilization studies in mice deficient in the C5 component of the ComC and found that these mice do not display circadian changes in the number of HSPCs circulating in PB [7, 17, 18]. We have also proposed that these changes in activation of the ComC can be explained by the hypoxia that occurs during deep sleep [5, 12, 19].

We have also reported [20, 21], as has been subsequently supported by other investigators [22–24], that the levels of two bioactive phosphosphingolipids, sphingosine-1-phosphate (S1P) and ceramide-1-phosphate (C1P), form steep chemotactic gradients between PB and BM for HSPCs residing in BM stem cell niches. It is well known that activation of the ComC may affect the level of S1P in PB, as an end product of ComC activation, the membrane attack complex (MAC, also known as C5b-C9), may increase release of this chemoattractant from erythrocytes and activated platelets [20].

Based on these observations, we became interested in circadian changes in ComC activation and the S1P level in PB. Accordingly, we measured the circadian changes in ComC activation by measuring the PB level of the more stable C3a and C5a anaphylatoxins cleavage fragments - C3a-desArg and C5a-desArg and also the final product of ComC activation, the MAC, by employing an enzyme-linked immunosorbent assay (ELISA). In parallel, we performed measurements of the PB level of S1P by employing reverse-phase high-performance liquid chromatography (RP-HPLC). PB was collected from 66 healthy volunteers at 8 am, 2 pm, 8 pm, and 2 am. To assess the normal circadian rhythm in our patients, we measured the PB levels of melatonin at the same time points.

Our results provide evidence for circadian changes in activation of the ComC–S1P axis, which, according to our hypothesis, drives circadian changes in the number of HSPCs circulating in PB [25]. These observations are also relevant to better understanding certain circadian-associated pathologies, such as the exacerbation of inflammatory symptoms at night, the onset of hemolysis in paroxysmal nocturnal hemoglobinuria (PNH) patients, and the prevalence of stroke incidence in the early morning hours [26].

## Materials and Methods

### Study Group

This study was conducted in a group of 66 healthy volunteers aged 20–50 years, divided equally by gender: 33 women (mean age 31 ± 7 years) and 33 men (mean age 34 ± 10 years). The volunteers knowingly and willingly signed consent to participate in the study, and each filled out a survey regarding their lifestyle before participating in the experiment. The survey contained questions about the state of health, nutrition, duration of sleep, and medicines and supplements used by the participants. The following exclusion criteria were applied: the use of contraceptives, hormone replacement therapy, intake of medicines used to treat chronic diseases and other pharmacological treatments, intake of aspirin and antibiotics within a month of the tests, pregnancy, and a multi-shift work schedule. Participants were provided with convenient conditions, similar to those in everyday life, with a room with beds for rest and sleep (which was especially important in the late evening and at night). The volunteers were waked up at 2 am and blood has been drawn immediately and after this procedure they returned to their regular sleep. This arrangement allowed maintenance of a normal waking–sleeping rhythm throughout the 24 h of the experiment. We believe that the application of appropriate conditions and inclusion and exclusion criteria ensured high credibility for the results obtained. The Bioethical Commission at the Pomeranian Medical University gave consent for all tests (No. KB-0012/99/14).

### Study Material

PB was collected four times from the basilic vein at equal 6-h intervals (2 am, 8 am, 2 pm, and 8 pm) by qualified medical personnel into a tube with K_2_EDTA (8 mL) and blood clot (8 mL). A complete blood count (CBC) of the whole blood was done using an ABX Micros 60 analyzer in K_2_EDTA to verify the health status of the tested group. The collected samples were then centrifuged (2600 rpm, 20 °C, 10 min) to separate plasma and serum. To the confirm health status of the group of subjects, additional determinations of basic biochemical parameters were also performed. These included glucose and a lipid panel (total cholesterol, triglycerides, low-density lipoproteins [LDL], high-density lipoproteins [HDL], total protein, albumin, creatinine, and uric acid). In addition, the concentration of mineral components such as organic phosphorus, total magnesium, and total calcium were determined. BioMaxima kits were used to make these determinations, and all analyses were carried out in accordance with the manufacturer’s recommendations. Any determinations deviating from reference values were additional criteria for excluding a participant from the study. The remaining material (both plasma and serum) was transferred to new tubes and frozen at −80 °C until the assays were performed. The stored plasma was used for the S1P assay, while the stored serum was used to determine melatonin and the concentration of components of the complement system (C3a, C5a, MAC) at all sampling times. In the case of the anaphylatoxins C3a and C5a, their breakdown products, C3a-desArg and C5a-desArg, were determined, because, under physiological conditions, both C3a and C5a are unstable and are very quickly converted by endogenous carboxypeptidase N in the serum to these less active but much more stable forms [27].

### Determination of Melatonin Concentration in Serum

To confirm preservation of the normal rhythm of sleeping and waking throughout the day, the concentration of melatonin in blood serum was determined four times. An ELISA reagent assay (Human Melatonin ELISA Kit, Cloud-Clone Corp.) was used for this analysis. All determinations were made in accordance with the guidelines provided by the manufacturer. The concentration of melatonin was calculated from a previously prepared in each individual ELISA plate standard curve based on serial dilutions of the standard solution included in the commercial kit. The absorbance was measured on an EnVision microplate reader (Perkin Elmer) at 450 nm.

### Determinations of C3a-desArg and C5a-desArg Concentration in Serum

To determine the serum concentrations of C3a-desArg and C5a-desArg, ELISA reagent kits were used (Human C3a ELISA Kit, BD OptEIA™ and Human C5a ELISA Kit II, BD OptEIA™). These kits contain plates with wells coated with monoclonal antibodies specific for human C3a-desArg and C5a-desArg, respectively. The determinations were made in accordance with the guidelines provided by the manufacturer. The concentrations of C3a-desArg and C5a-desArg were calculated from a previously prepared in each individual plate standard curve based on serial dilutions of the freeze-dried standard solution included in each commercial kit. The absorbance was measured on an EnVision microplate reader (Perkin Elmer) at 450 nm.

### Determination of the MAC Concentration in Serum

The ELISA reagent kit (Human C5b-9 ELISA Set, BD OptEIA™) was used to determine the serum MAC concentration. The determinations were made in accordance with the guidelines provided by the manufacturer. The MAC concentration was calculated from a previously prepared in each individual plate standard curve based on serial dilutions of the standard solution included in the commercial kit. The absorbance was measured on an EnVision microplate reader (Perkin Elmer) at 450 nm.

### Determination of Plasma and Serum S1P Levels by Reverse-Phase High-Performance Liquid Chromatography (RP-HPLC)

Plasma/serum and the internal standard C17-S1P (Avanti Polar Lipids) used in this experiment were brought to room temperature. Plasma/serum (100 μL) and 30 μL of synthetic C17-S1P standard in methanol (MetOH):10 mM K_2_HPO_4_ (9:1, *v*/v), pH 7.2, were added to the glass tube. Under the same conditions a mixture containing C17-S1P and C18-S1P was prepared. Samples were vortexed, and 1 M NaCl added to obtain 1 mL. Subsequently, 1 mL of MetOH, 300 mL of concentrated HCl, and 2 mL of chloroform were added. Each step was preceded by mixing with a vortex. The samples were mixed on a test tube rotator for 20 min, and centrifuged (3500 rpm, 20° C, 3 min). The lower organic phase was withdrawn and transferred to a new tube. The upper layer was re-extracted by adding 2 mL of chloroform, mixing on the test tube rotator for 10 min, and recentrifuging. The lower organic phase containing S1P was combined with the previous lower layer. The samples were then dried in a vacuum centrifuge (RVC 2–25 CD) at 45 °C for 45–60 min. The dried extracts were stored at −80 °C until analysis. Before measurement, the extracts were brought to room temperature and dissolved in 130 μL methanol and 20 μL ortho-phthalaldehyde (OPA). Simultaneously, a mixture was prepared consisting of 30 μL of C17-S1P, 30 μL of C18-S1P, and 940 μL of MetOH:K_2_HPO_4_ (9:1, *v*/v), pH 7.2, from which 600 μL was taken, transferred to a new sample tube, and 75 μL of OPA added. All samples with OPA were incubated for 20 min at room temperature in a dark place and then centrifuged (6000 rpm, 20 °C, 10 min). The supernatant was transferred to a new sample tube, and 20 μL of 10 mM K_2_HPO_4_ buffer, pH 7.2, was added. After centrifugation the mixture was immediately transferred to a clean bottle. Buffer samples were incubated for 10 min at +4 °C and then centrifuged again (6000 rpm, 20 °C, 10 min). After centrifugation, the clear supernatant was transferred to a clean bottle and RP-HPLC was performed. Chromatographic data was developed using HP Chemstation software (Agilent, USA). A C18-ARII Cosmosil 5-μm C18-ARII column (150 × 4.6) at 25 °C and a 5-μm C18-ARII pre-column (10 × 4.6, Waters) were used for separation in the reverse phase. An isocratic method with a mobile phase consisting of 10 mM K_2_HPO_4,_ pH 5.5, and methanol (15:85, *v*/v) was used. Samples of 50 μL were injected on the column every 30 min at a flow rate of 1 mL/min. The wavelength for detecting S1P derivatives was 340 nm for excitation and 455 nm for emission. S1P concentration was calculated on the basis of the peak surface area of the internal standard C17-S1P.

### Statistical Analysis

The serum concentration of melatonin was determined to confirm the correct rhythm of sleeping and waking, which was followed by determinations of the components of the complement system in serum (C3a, C5a, MAC) and plasma/serum S1P concentrations. The distribution of these factors was assessed using a Kolmogorov–Smirnov test. It was shown that the tested parameters differed from a normal distribution, and so in order to determine whether the concentrations of melatonin, components of the complement system (C3a, C5a, MAC), and S1P were affected by a circadian rhythm, the nonparametric Friedman ANOVA test and Kendall’s coefficient of concordance were used. A Wilcoxon signed-rank test was used to directly assess the differences in concentration of melatonin, components of the complement system (C3a, C5a, MAC), and S1P at each time point. In all cases, these tests were performed taking into account the sex of the volunteers tested. The results were prepared using Statistica PL 13 (StatSoft, Poland) and MS Excel 2013 software.

## Results

### Influence of Circadian Rhythm on Changes in Melatonin PB Serum Concentration

**Fig. 1:**
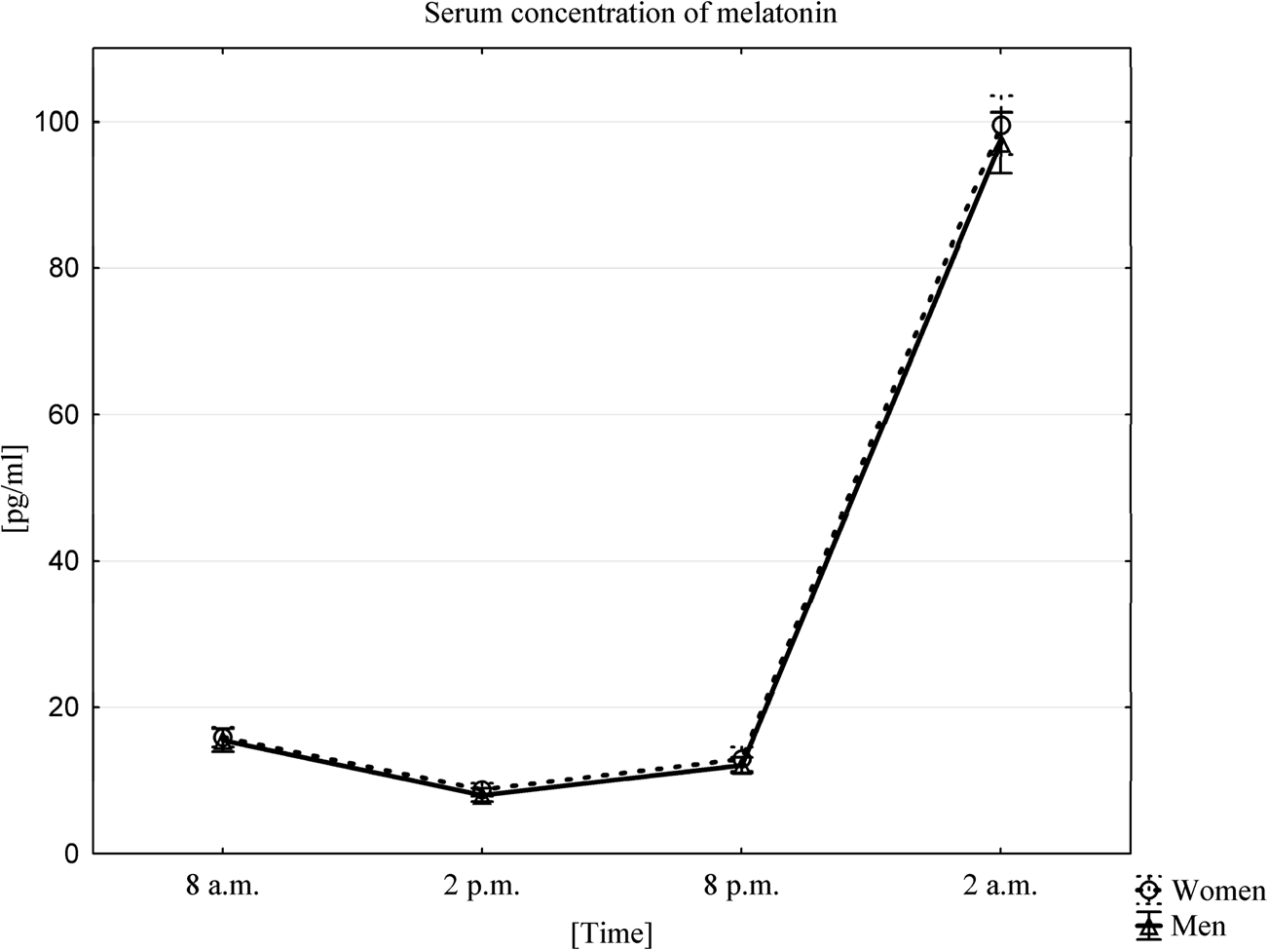
Average serum concentration of melatonin in women (*n* = 33) and men (n = 33) at different sampling time points, data presented as means ±95% Confidence Interval. The occurrence of a circadian melatonin rhythm was confirmed using a Friedman ANOVA test and Kendall’s coefficient of concordance for both women and men. A Wilcoxon signed-rank test confirmed the presence of statistically significant differences between all time points (*p* < 0.0001), both in men and in women

In our study, the average serum concentration of melatonin, for both women and men, was highest in the 2 am samples (99.52 ± 11.25 pg/mL in women, 97.12 ± 11.73 pg/mL in men). The lowest concentrations of this hormone were observed 12 h later in the 2 pm samples (8.7 ± 2.53 pg/mL in women, 7.97 ± 2.61 pg/mL in men) (Fig. 1).

### Influence of Circadian Rhythms on Changes in C3a-desArg and C5a-desArg Concentration in PB Serum

**Fig. 2:**
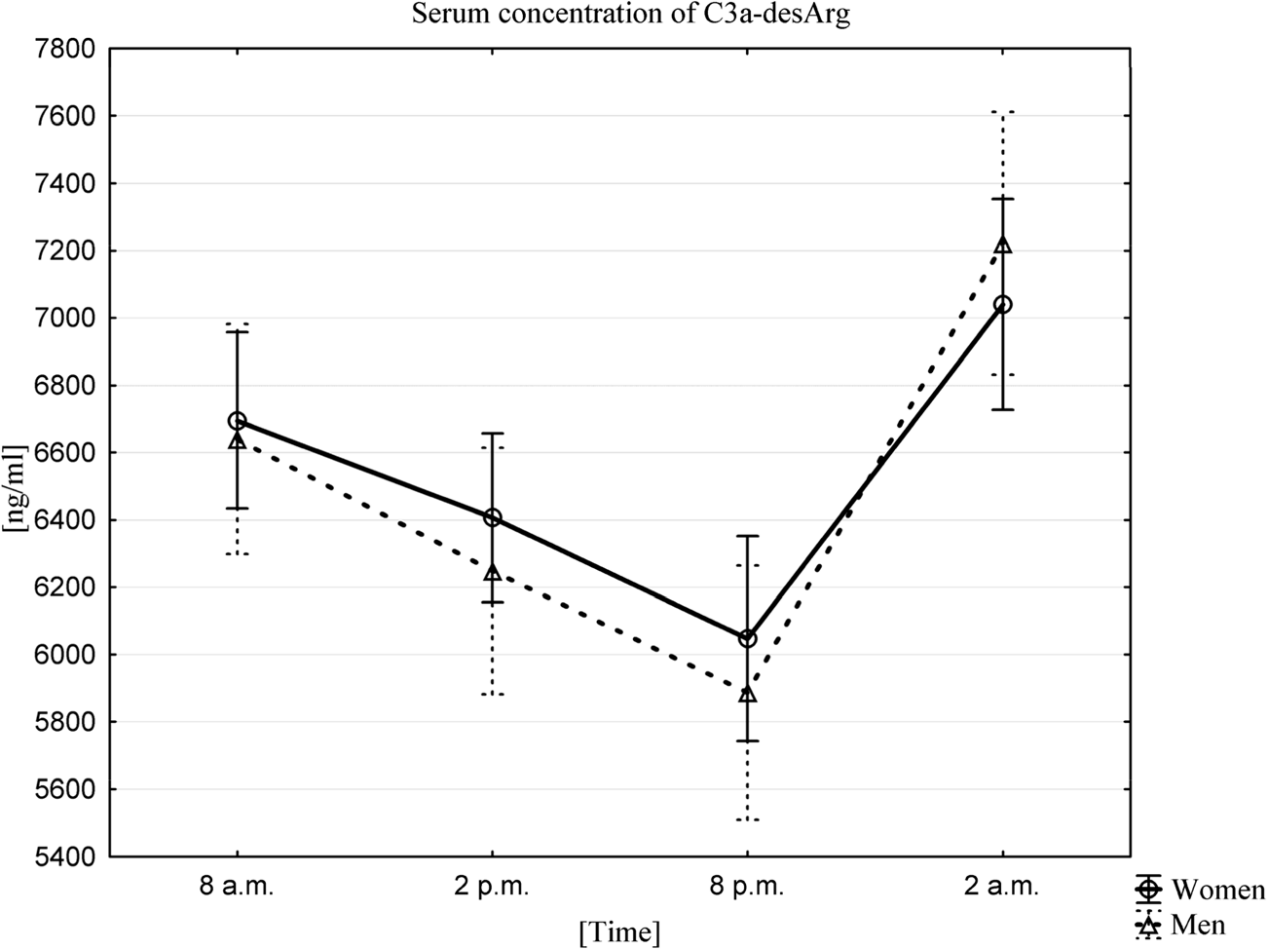
Average serum concentration of C3a-desArg in women (n = 33) and men (n = 33) at different sampling time points, data presented as means ±95% Confidence Interval. Friedman ANOVA and Kendall’s coefficient of concordance (p < 0.0001)

The average serum concentration of **C3a-desArg** in women and men was highest at 2 am (7040.0 ± 882.5 ng/mL in women, 7221.5 ± 1099.7 ng/mL in men). By contrast, the lowest serum concentrations for this anaphylatoxin were observed 18 h later, at 8 pm (6047.2 ± 859.3 ng/mL in women, 5886.8 ± 1065.8 ng/mL in men). Statistical analysis confirmed the existence of a C3a-desArg circadian rhythm using a Friedman ANOVA test and Kendall’s coefficient of concordance in both women and men (Fig. 2). A Wilcoxon signed-rank test confirmed that there were statistically significant differences between the values at all time points (*p* < 0.0001).

**Fig. 3:**
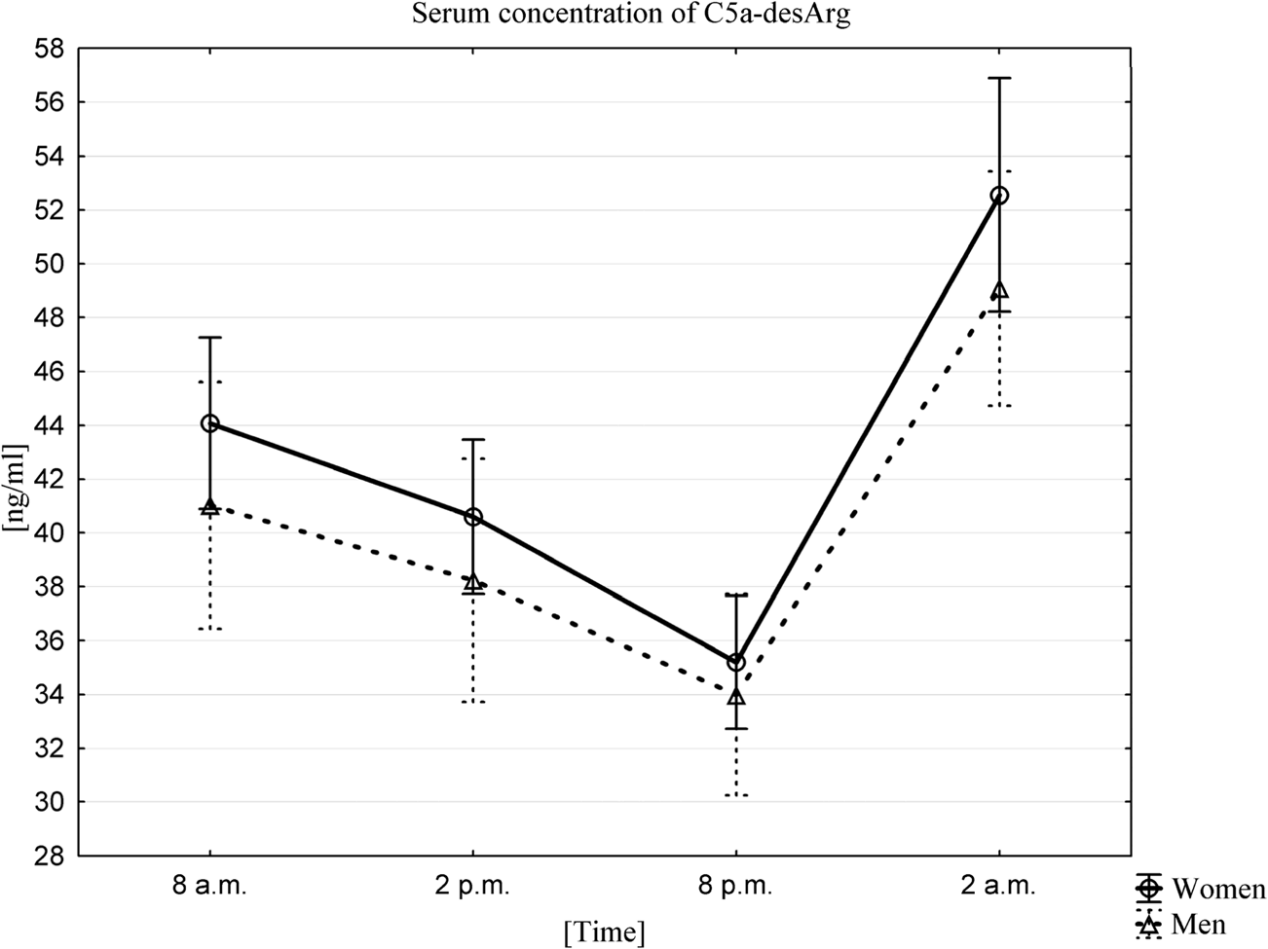
Average serum concentration of C5a-desArg in women (n = 33) and men (n = 33) at different sampling time points, data presented as means ±95% Confidence Interval. Friedman ANOVA and Kendall’s coefficient of concordance (p < 0.0001)

Similarly the average serum concentration of C5a-desArg, both in women and men, was highest at 2 am (52.6 ± 12.2 ng/mL in women, 49.1 ± 12.3 ng/mL in men). Again, the lowest serum concentrations of this anaphylatoxin were observed 18 h later, at 8 pm (35.2 ± 7.0 ng/mL in women, 34.0 ± 10.5 ng/mL in men). Similarly, a circadian rhythm of plasma C5a-desArg was confirmed by a Friedman ANOVA test and Kendall’s coefficient of concordance both in women and men (Fig. 3). A Wilcoxon signed-rank test confirmed that there were statistically significant differences between the values at all time points (*p* < 0.0001).

### Influence of a Circadian Rhythm on Changes in MAC Concentration in PB Serum

**Fig. 4:**
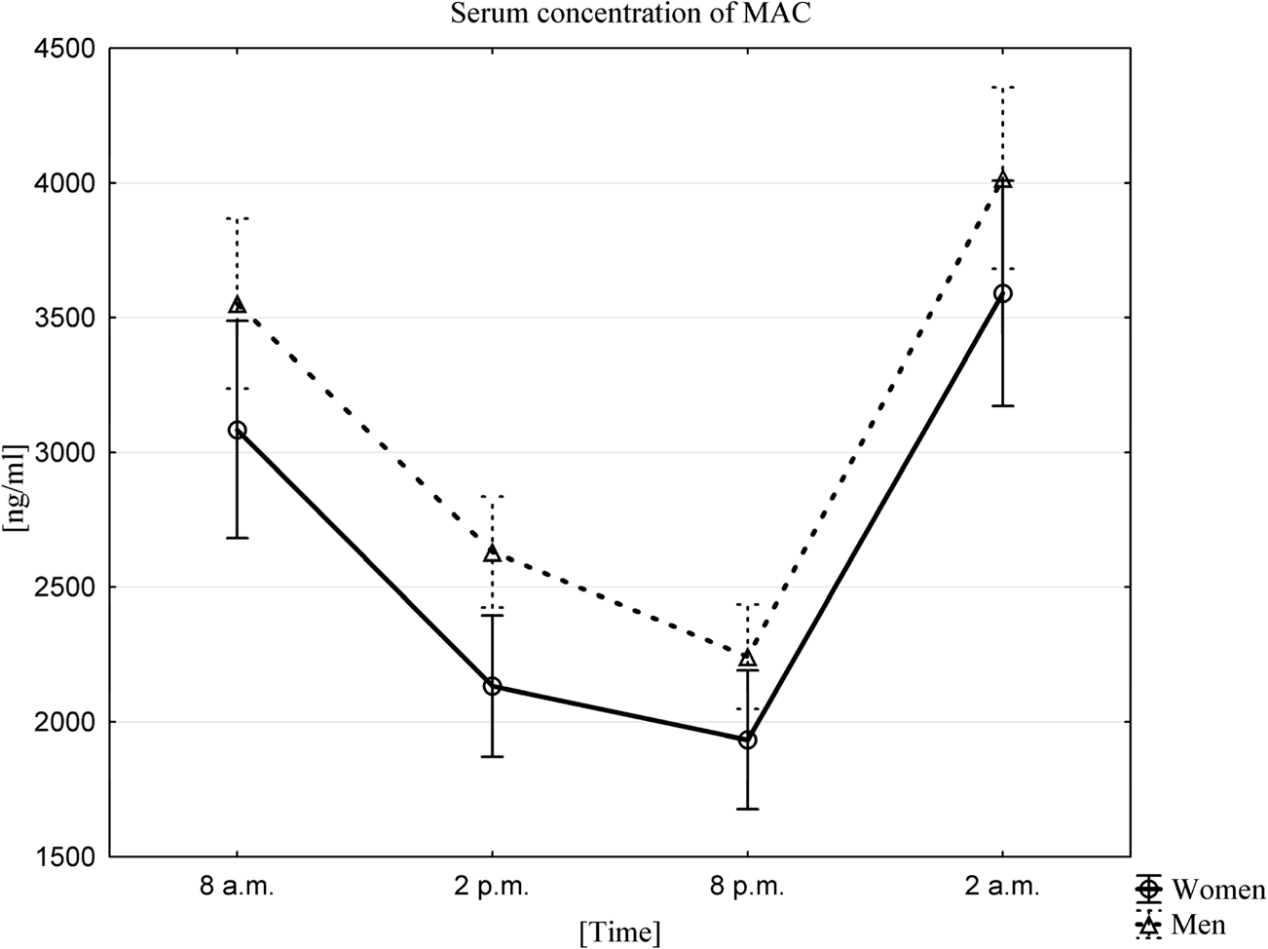
The average serum MAC concentration in women (n = 33) and men (n = 33) at different sampling time points, data presented as means ±95% Confidence. Friedman ANOVA and Kendall’s coefficient of concordance (p < 0.0001)

The average serum MAC concentration in both women and men was highest at 2 am (3590.6 ± 1108.0 ng/mL in women, 4018.2 ± 949.7 ng/mL in men) and lowest 18 h later, at 8 pm (1933.6 ± 727.2 ng/mL in women, 2242.9 ± 547.4 ng/mL in men). The existence of a MAC circadian rhythm was confirmed using a Friedman ANOVA test and Kendall’s coefficient of concordance in both women and men (Fig. 4). A Wilcoxon signed-rank test confirmed that there were statistically significant differences between the values at all sampling times (*p* < 0.0001).

### Influence of a Circadian Rhythm on Changes in Plasma S1P Concentration

**Fig. 5:**
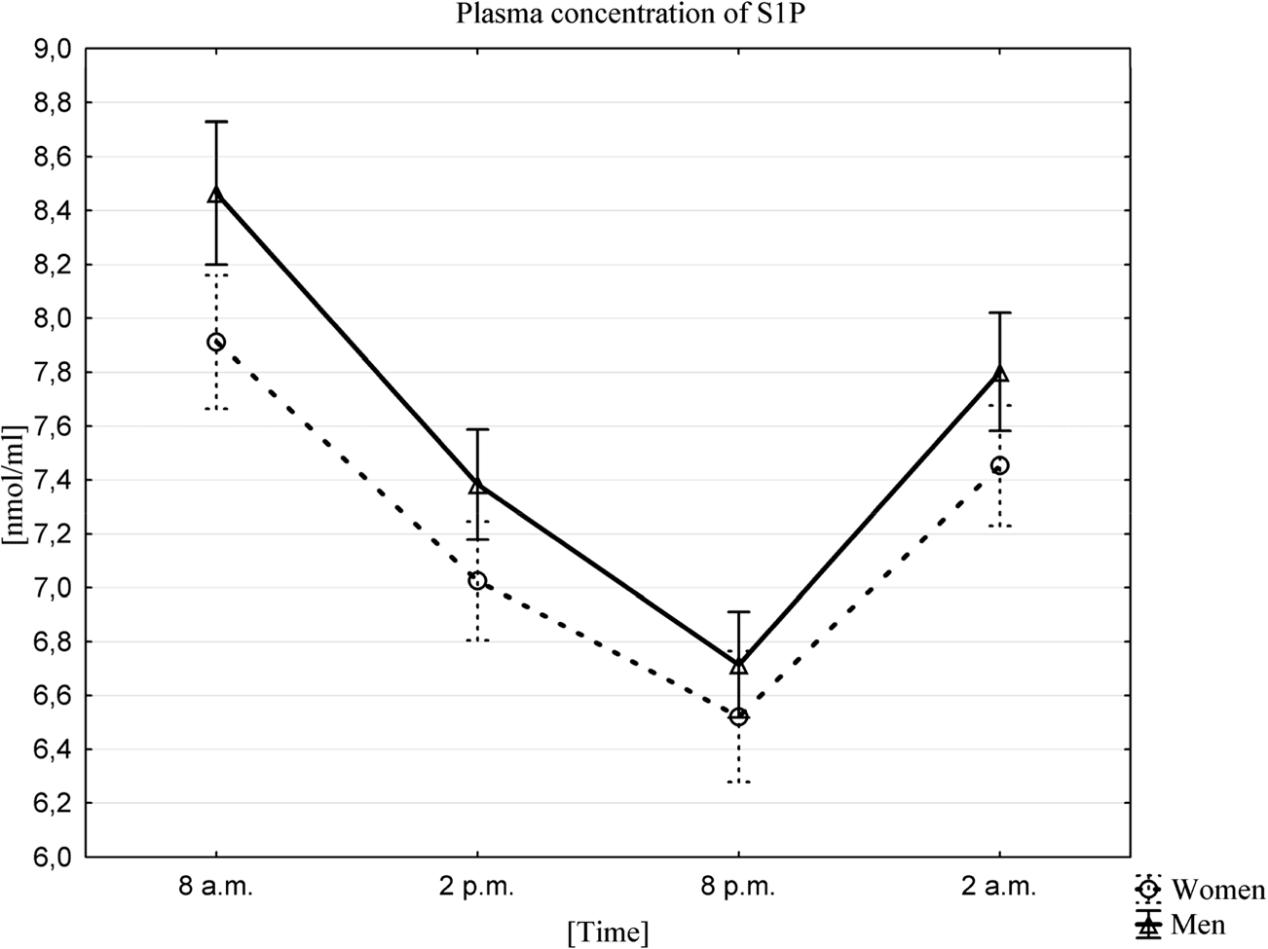
Mean plasma concentration of S1P, in women (n = 33) and men (n = 33) at different sampling time points, data presented as means ±95% Confidence Interval. Friedman ANOVA and Kendall correlation coefficients (p < 0.0001)

The highest mean plasma concentrations of S1P for both sexes occurred at 8 am (7.91 ± 0.71 nmol/mL in women, 8.46 ± 0.75 nmol/mL in men), and the lowest at 8 pm (6.52 ± 0.69 nmol/mL in women, 6.71 ± 0.55 nmol/ml in men). The existence of a circadian rhythm in plasma S1P in men and women (Fig. 5) was confirmed by Friedman ANOVA and Kendall correlation coefficients. The Wilcoxon matched-pairs, signed-rank tests showed statistically significant differences between the values at all time points. The concentrations between the time points tested in both men and women showed a statistically significant correlation at *p* < 0.0001.

## Discussion

The seminal observation of this work is our demonstration of circadian activation of the ComC–S1P axis in normal healthy volunteers. This observation has implications for circadian release or mobilization of HSPCs from BM into PB, because activation of the ComC in the BM microenvironment is the driving force for release of these cells from their BM niches, and this occurs in the presence of an S1P gradient between BM and PB [25].

The occurrence of a circadian rhythm in living organisms can be assessed by measuring the levels of melatonin, a hormone released into PB in a 24-h cycle [28]. Therefore, we followed circadian changes in melatonin level in all our volunteers and, as expected, observed the highest concentration of melatonin at night, at around 2 am, and the lowest during the day, at around 2 pm [5, 28–30]. These results confirmed that our experiments were designed well and preserved the normal sleep of volunteers and an unperturbed circadian rhythm.

Activation of the ComC, which was the main subject of our study, is a crucial part of the innate immunity system, which consists of over 50 circulating or cell surface-bound proteins (zymogens, effectors, receptors, or control proteins) [31]. Complement proteins are synthesized mainly by hepatocytes, but they can also be locally produced in peripheral tissues and circulate as inactive precursors, and their activation occurs in a cascade type of reaction in which the preceding cleavage product cleaves and activates the next component in the cascade [32]. The physiological role of the ComC is as a defense against pyrogenic bacterial infection and the removal of immune complexes and inflammatory cell damage products [33]. In addition to regulating the mobilization of HSPCs [34], it affects the coagulation system [35], angiogenesis [36], tissue regeneration [37] and lipid metabolism [38]. In addition, the C3 and C5 complement component fragments, the C3a and C5a anaphylatoxins, have been shown to stimulate the secretion of adrenocorticotropic hormone (ACTH), growth hormone (GH) and prolactin (PRL), hormones with well-known circadian regulation [5]. This crosstalk between the innate immunity system and the hypothalamic–pituitary–adrenal axis is an important regulator of the inflammation process through the modulation of glucocorticoid secretion [6].

The ComC is activated by the classical-, alternative-, and mannan-binding lectin pathways [34]. This latter pathway plays a crucial role in induction of the “sterile inflammation state” in the BM microenvironment during pharmacology-induced mobilization of HSPCs [39]. The egress of HSPCs into PB is subsequently the result of a steep S1P gradient between BM and PB. S1P is a bioactive phosphosphingolipid that is a major chemoattractant for HSPCs present in circulating blood [20] and lymph [40].

Circadian activation of the ComC, with its peak during deep sleep, has been proposed very recently and has been supported by demonstrating a decrease in the levels of C3 and C4 ComC components at night and an increase in the C3 cleavage fragment, the anaphylatoxin C3a. Interestingly, these changes in ComC component levels occurred only in patients in a normal sleep rhythm [5]. In our previous work we demonstrated that mice that are deficient in the C5 component of the ComC do not show a circadian release of HSPCs into PB [7, 17, 18] and proposed that activation of the ComC as a result of the hypoxia that occurs during deep sleep at night is most likely required to maintain a circadian rhythm in stem cell circulation [5, 12, 19].

Activation of the ComC at night has two important clinical implications. First, because of the presence of crosstalk between the ComC and the coagulation cascade (CoaC) [34], ComC cleavage fragments may activate platelets, which may explain the incidence of stroke or sudden cardiac death in the early morning hours [26]. Activation of the ComC during the night is also demonstrated in patients suffering from paroxysmal nocturnal hemoglobinuria (PNH), in which hemolysis of red blood cells occurs at the nocturnal peak of ComC activation [41].

Our results confirm that the ComC undergoes circadian activation and demonstrate that the ComC is activated by release of the potent anaphylatoxin C5a as well as generation of the MAC, the terminal lytic product of ComC activation. The MAC is than responsible for additional release of S1P from red blood cells as well as for activation of blood platelets [20].

Another important observation reported in the current paper is the presence of a circadian rhythm in the sphingosine-1-phosphate (S1P) level in PB, with a morning peak at 8 am. In recent years, considerable progress has been made in understanding the role of S1P in several physiological and pathological processes [42]. S1P regulates motility, gradient-dependent migration, growth, and the survival of several cell types [43] and plays a role in the egress of HSPCs from BM into PB [20] and lymph [40], the circulation of lymphocytes, and angiogenesis [43]. Despite the importance of S1P, no information was previously available about its circadian rhythm in human PB. Thus, our study fills this gap and shows that the peak in S1P level in the morning hours may direct circadian egress of HSPCs into PB [44]. The increase in S1P may be a result of the ComC and its subclinical lytic effect on erythrocytes and activation of platelets, as erythrocytes and platelets are important carriers of this bioactive phosphosphingolipid [45–49]. Other rich sources of S1P in the circulation are albumin and high-density lipoproteins (HDLs) [49–51], which may potentially release S1P in response to increases of proteolytic and lipolytic enzymes in PB in the early morning hours. However, this possibility requires further study. It would be also important to see if similar mechanisms regulates circadian release of other stem cells residing in BM tissue [52–56].

Corroborating our circadian S1P level results, Chua and coworkers also demonstrated the presence of a circadian rhythm for two sphingolipids, sphingosine and ceramide, which are precursors in S1P synthesis. It was found that these sphingolipids also exhibit a circadian rhythm, reaching their highest concentrations at around 6 pm, with the lowest concentration at around 6 am [57]. This “reverse” circadian rhythm of sphingosine and ceramide, which are almost perfectly out of phase with the S1P level in PB, can be explained by a decrease in these substrates during S1P synthesis, which increases even as the concentrations of these precursors decrease [58]. It is likely that this could be accompanied by circadian regulation of enzymes involved in S1P synthesis, which may lead to a reduction of sphingosine and ceramide levels and to an increase in their product, S1P, but so far studies of this matter have not been undertaken.

In conclusion, we demonstrated the presence of circadian changes in activity of the ComC–S1P axis. However, while we did not measure circadian release of HSPCs into PB in the current study, our results support the concept that this axis plays an important role in circadian egress of HSPCs into circulation [16]. Since egress of HSPCs from BM into PB, as demonstrated in other elegant studies, is also regulated by circadian tonus of the adrenergic system, further studies are needed to study potential crosstalk between the ComC–S1P axis and adrenergic signaling in BM.

Determination of the changes in circadian regulation of ComC and S1P level in PB shed more light on regeneration processes that occur during sleep and explain better the cellular mechanisms underlying the regulation of the immune system and its impact on the stem cell compartment. Our research may be useful in the prognosis and diagnosis of diseases in which disorders of the circadian rhythm would affect the activation of the complement system and in consequences S1P release into PB. In the era of dynamic development of regenerative medicine these are issues of considerable importance, and may be helpful in improving the therapeutic strategies, especially in patients in whom the implemented treatment disrupts the activation of the complement cascade and consequently affects stem cell trafficking.
